# Investigation of the Thermal Conductivity of Resin-Based Lightweight Composites Filled with Hollow Glass Microspheres

**DOI:** 10.3390/polym12030518

**Published:** 2020-02-29

**Authors:** Zhipeng Xing, Hongjun Ke, Xiaodong Wang, Ting Zheng, Yingjie Qiao, Kaixuan Chen, Xiaohong Zhang, Lili Zhang, Chengying Bai, Zhuoran Li

**Affiliations:** College of Materials Science and Chemical Engineering, Harbin Engineering University, Harbin 150001, China; 13945158401@163.com (Z.X.); kehongjun2012@126.com (H.K.); qiaoyingjie99@163.com (Y.Q.); chen.kx.fw@gmail.com (K.C.); zhangxiaohong@hrbeu.edu.cn (X.Z.); zhanglili1984@hrbeu.edu.cn (L.Z.); chengyingbai@163.com (C.B.); lizhuoran777@gmail.com (Z.L.)

**Keywords:** lightweight composites, thermal insulation composites, HGMs, thermal conductivity

## Abstract

The design and development of thermal insulation materials is very important for the treatment of offshore oil pipelines. Understanding thermal energy transport in thermal insulation materials and predicting their thermal conductivities have important theoretical and practical value for the design of thermal insulation materials. In this work, lightweight and thermally insulated (LWTI) composites with the desired mechanical strength for offshore oil pipelines applications were prepared using epoxy resin (EP) as the matrix and hollow glass microspheres (HGMs) as the filler. The morphology, density, and mechanical properties of HGM/EP LWTI composites were studied first. The flexural strength and the flexural modulus of HGM/EP LWTI composites could still be as high as 22.34 ± 2.75 Mpa and 1.34 ± 0.03 GPa, respectively, while the density was only 0.591 g/cm^3^. The relationship between the effective thermal conductivity of HGM/EP LWTI composites and material parameters (sizes and contents together) has been studied systematically. A three-phase prediction model was built using the self-consistent approximation method to predict the effective thermal conductivity of HGM/EP LWTI composites, and the resin matrix, the wall thickness, the HGM particle size, and other parameters (such as air) were fully considered during the derivation of this three-phase thermal conductivity model. Finally, the insulation mechanism of HGM/EP LWTI composites was systematically analyzed. The thermal conductivities of HGM/EP LWTI composites with different diameters and HGM contents calculated by the three-phase prediction model agreed well with the experimental test results, with a minimum error of only 0.69%. Thus, this three-phase thermal conductivity model can be used to theoretically simulate the thermal conductivity of epoxy resin-based LWTI composites and can be the theoretical basis for the design and prediction of the thermal conductivity of other similar hollow spheres filled materials.

## 1. Introduction

Onshore and shallow sea hydrocarbon resources are being exhausted gradually owing to excessive exploitation. Consequently, deep-sea hydrocarbon resource development has attracted increasing attention from the oil industry in recent years [1,2,3]. In addition, offshore oil transport involves certain unique features, such as long transmission distances, low water temperature, and complex crude oil components [4,5]. During long-distance transportation in cool seawater, crude oil can easily form wax or icy crystals and precipitate on oil pipeline shells, which will decrease fluidity, cause blockage, and eventually produce an accident. Hence, some treatments for oil pipelines are necessary to be done for maintaining temperatures at the optimal condition inside the pipelines. There are different methods to achieve the purpose above, but use of thermal insulation materials outside of oil pipelines is one of the most convenient. Thus, the design and development of thermally insulated materials is very important for the field of marine engineering [6,7].

Conventional insulation materials, including natural materials, inorganic fiber materials, and organic foam materials, have been widely used [2]. Among these, polymer foams are a microporous material made from polymer matrix filled with gas, which presents low density, low water absorption, high porosity, and excellent insulation performance. It could be a suitable alternative to overcome the technical limitations of traditional structural alloys for deep-water oil pipelines owing to their high strength, along with their reduced density compared with metals [8]. The polymer foams made by epoxy are a major development of syntactic foams, which could be considered for composite structural application as they show good adhesion behavior on many substrates, excellent mechanical properties, and high stability. However, pure epoxy foams always present high brittleness and a high pulverization ratio when low density is required for oil pipelines applications, and are also not sufficient for oil pipelines under stringent thermal insulation. The prevailing technique to improve the capacity of polymer foam to inhibit heat transmission and maintain certain mechanical properties is to fabricate composites by introducing additional thermally insulated fillers or many interfaces into the final materials [9,10]. In theory, hollow microspheres (e.g., hollow glass spheres, hollow ceramic spheres, hollow plastic spheres) have distinct advantages, including a low thermal conductivity coefficient and low density, because they contain air or other gas inside [11,12,13]. Moreover, hollow microspheres are introduced in the resin before curing in order to generate controlled porosity distribution at the same time. They are, therefore, the ideal thermal insulation filler to modify polymer resins in industry. Y. Liao et al. [14] studied the thermophysical property of hollow silica spheres by the experimental test, theoretical calculation, and finite element simulation, and found that the silica hollow spheres were highly efficient heat-insulating materials. S.N. Patanka et al. [15] developed the high crushing strength and low thermal conductivity polyethylene composite by mixing hollow glass spheres in polymers system. On the other hand, the thermal behavior of materials can be evaluated by the effective thermal conductivity [16,17]. Understanding thermal energy transport in materials and predicting the thermal conductivity of composites can benefit the design of a composite system and material structure optimization to achieve ideal material thermal insulation properties for oil pipelines insulation applications [18]. Various theoretical studies and numerical methods have been proposed to study the effective thermal conductivity of composite materials [19], including effective medium theory, the finite element method [20], the lattice Monte Carlo method [1], and the Maxwell approach [21]. Liao, et al. [20] used silica hollow spheres and polyurethane to develop an excellent thermal insulation film. The authors used the finite element simulation to study the compatibility between silica hollow spheres and polyurethane and calculate the thermal conductivity of the composite. G. Gedler et al. [22] developed polycarbonate–graphene nanocomposite foams and investigated their thermal conductivity, where a three-phase model (gas, polymer matrix, and filler) and a two-phase model (gas phase and composite phase) were used for calculating the thermal conductivity of foam materials. The results showed that thermal conductivity values displayed a better fit for a three-phase model. Among all effective thermal conductivity study methods above, mathematical methods offer a simple way of modeling specific geometries and phenomena, which can act as alternatives to the traditional finite element analysis and analytical approach. However, most studies’ thermal conductivity mathematical models involve solid-particle-filled composites or other polymer matrix composites [14,23,24], while reports of the mathematical models on microsized hollow glass-sphere-filled epoxy resins are relatively scarce. Thus, a thermal conductivity mathematical model that is more accurate and close to the hollow glass microsphere (HGM)/epoxy resin (EP) actual construction should be developed for the application of deep-sea oil pipelines. Moreover, the relationship between the effective thermal conductivity of HGMs filled polymer composites and material parameters (sizes and contents together) should also be studied systematically.

In this paper, lightweight and thermally insulated (LWTI) composites were prepared by using epoxy resin polymer as the matrix and HGMs as the thermal insulation filler. The relationship between the effective thermal conductivity of HGM/EP LWTI composites and material parameters was studied systematically. A three-phase model was built to predict the effective thermal conductivity of HGM/EP LWTI composites, and these prediction results were compared with the experimental data to systematically analyze the insulation mechanism of HGM/EP LWTI composites. In addition, the morphology, density, and mechanical properties of HGM/EP LWTI composites were also investigated.

## 2. Materials and Methods 

### 2.1. Materials

Epoxy resin (E51) with a density of 1.18 g/cm^3^ was purchased from Sinopec Baling Petrochemical Co., Ltd. (Yueyang, China). Methyl Tetrahydrophthalic Anhydride (MTHPA) (Zhejiang Alpharm Chemical Technology Co., Ltd, Zhengjiang, China) was used as a hardener. 3-Triethoxysilylpropylamine (KH550) was purchased from Yaohua chemical plant (Shanghai, China). The HGMs (HGMs) were provided by 3M China Ltd (Shanghai, China) and their properties are listed in Table S1.

### 2.2. Preparation of HGM/EP LWTI Composites

HGM/EP LWTI composites were prepared by mixing epoxy resin with HGMs. Different diameters (30, 40, and 55 μm) and contents (20 vol.%, 30 vol.%, 40 vol.%, and 50 vol.%) were selected for comparison, as shown in Table S2. The HGM/EP LWTI composites were fabricated according to the following steps: (i) First, the HGMs were modified using an ethanol solution containing 1 wt.% of KH550 coupling agent. Then, the modified HGMs were placed in an oven at 80–100 °C for 4–6 h to remove excess solvents. (ii) The epoxy resin and curing agent were mixed proportionally and then put into the vacuum oven to defoam. (iii) According to Table S2, the modified HGMs were poured into the resin-curing agent mixture. A blender was used to fully mix the HGMs with the epoxy resin and curing agent. The mixing speed was carefully controlled to ensure that the HGMs were fully mixed with the epoxy resin and not destroyed. (iv) The homogeneous mixture was poured into the mold for hot compression molding and cured at 50 °C for 1 h, 60 °C for 2 h, and 70 °C for 3 h, successively. (v) After curing, the mold was cooled to room temperature naturally before demolding. The obtained specimens were processed into the required size for testing.

### 2.3. Characterization and Measurements

The morphology and microstructure of HGM/EP LWTI composites were observed by scanning electron microscopy (SEM, S-4700, Hitachi Ltd., Tokyo, Japan) with an operating voltage of 10 kV. The density of HGM/EP LWTI composites was measured according to ISO 845. The theoretical density of HGM/EP LWTI composites with different designed constituent proportions can be calculated using Equation (1):

(1) $$\rho_{th}=\rho_{HGM}\emptyset_{HGM}+\rho_{m}\left(1-\emptyset_{HGM}\right)$$

where *ρ_th_* is the theoretical density, $\rho_{HGM}$ is the HGM density, *ρ_m_* is the density of the epoxy resin, and $\emptyset_{HGM}$ is the HGM volume fraction.

The void volume fraction ($\emptyset_{v}$) of the composites can be calculated using Equation (2):

(2) $$\varphi_{v}=\frac{\rho_{th}-\rho_{\exp}}{\rho_{th}}$$

where *ρ_exp_* is the measured density. The samples were cut into the standard dimension of 10 × 10 × 10 mm. Five specimens for each sample were tested in series to obtain the final results.

The flexural properties of 80 × 10 × 4 mm sized HGM/EP LWTI composites were tested according to the ASTM D790-07 flexural testing standard. An Instron 4505 universal testing machine was used to test and record data at room temperature using the static three-point bending load mode. Five specimens for each sample were tested in parallel to obtain the average value. The calculation of the flexural strength of composites is shown in Equation (3):

(3) $$R=1.5F_{R}\times \frac{L}{bd^{2}}\times 10^{6}$$

where *F_R_* is the maximum load; *L* is the spacing between the two supports; and *b* and *d* are the specimen width and thickness, respectively.

The flexural modulus is calculated according to Equation (4):

(4) $$E=\frac{L^{3}}{4bd^{3}}\times \frac{F_{t}}{x_{t}}\times 10^{6}$$

where *x_t_* is the deformation and F_t_ is the corresponding load.

## 3. Results

### 3.1. The Morphology and Density of HGM/EP LWTI Composites

**Figure 1:**
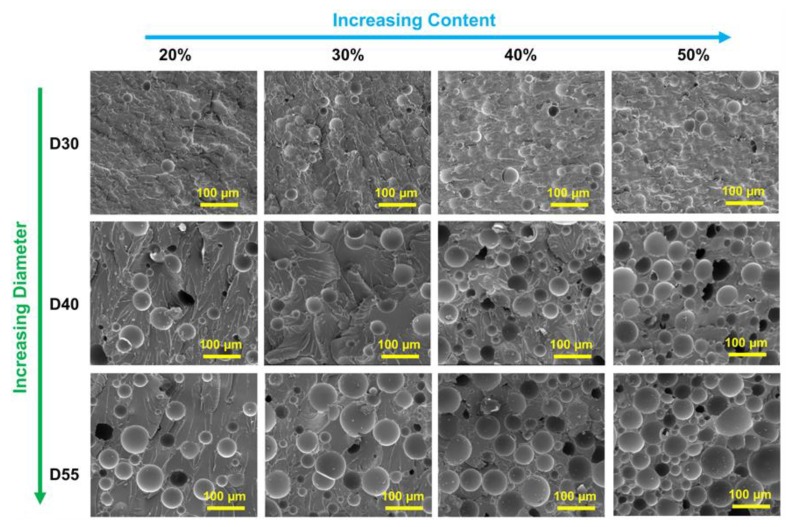
Scanning electron microscopy (SEM) graphs of lightweight composites with different hollow glass microsphere (HGM) diameters and contents.

To evaluate the HGM distribution in the epoxy resin, the morphology and microstructure of HGM/EP LWTI composites with different HGM diameters were observed using SEM, as shown in Figure 1. The HGMs were exposed on the surface and evenly dispersed throughout the resin matrix. There was an obvious increase in voids in the composites as the HGM diameter and volume percentage increased from 30 μm to 55 μm, and 20% to 50%, respectively. The theoretical density, measured density, and void volume fraction of the 12 groups of composites with different HGM sizes and volume fractions are listed in Table 1.

Table 1 shows that, for the same HGM volume fraction in the HGM/EP LWTI composites, the density decreased noticeably and the void volume fraction increased with the increasing HGM diameter. As the diameter increased from 30 μm to 55 μm, the density of the HGM/EP LWTI composites with 20 vol.%, 30 vol.%, 40 vol.%, and 50 vol.% of HGMs decreased from 1.045 g/cm^3^ to 0.930 g/cm^3^, 0.979 g/cm^3^ to 0.826 g/cm^3^, 0.912 g/cm^3^ to 0.709 g/cm^3^, and 0.845 g/cm^3^ to 0.591 g/cm^3^, respectively. The theoretically calculated density values and the experimental test results for the HGM/EP LWTI composites showed the same change behavior, but the theoretically calculated values were slightly higher than the test results, owing to residual bubbles during the preparation steps including mechanical mixing, molding, and curing of the EP and HGMs. From the void volume fraction calculation, when the HGM content is 20 vol.%, the void volume fraction of HGM/EP LWTI composites increases from 2.41% to 5.18% as the HGM diameter increases from 30 μm to 55 μm. The HGM/EP LWTI composites with 30 vol.% HGMs increased from 3.2% to 5.86% and further increased from 4.33% to 8.39% and 5.50% to 11.71% for HGM/EP LWTI composites with 40 vol.% HGMs and 50 vol.% HGMs, respectively. As the HGM content increases, EP may not surround the HGM very well, resulting in increased gap formation in the composites. On the other hand, the increasing HGM content also makes it more difficult to mix the HGMs with the EP, and insufficient resin mixture can cause the HGMs to stick together, and bubbles to form in the composite.

### 3.2. The Flexural Properties of HGM/EP LWTI Composites

**Figure 2:**
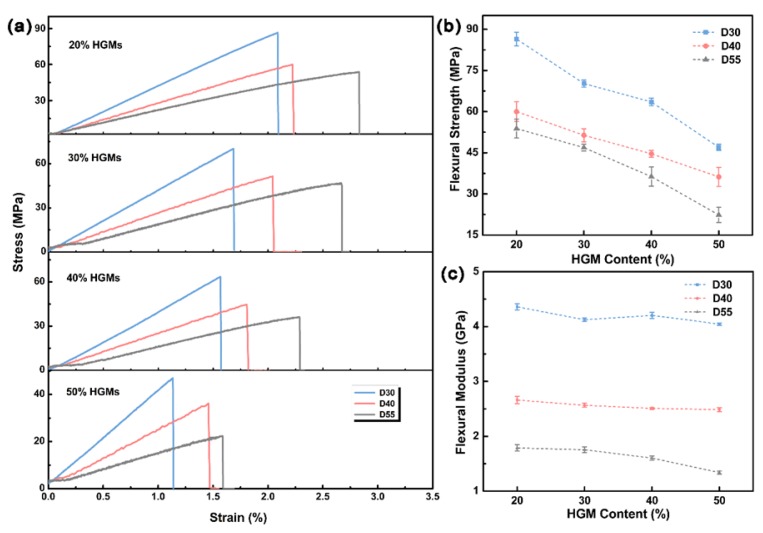
The flexural properties of HGM/EP LWTI composites with different HGM diameter and content. (**a**) bending stress–strain curves; (**b**) the flexural strength; (**c**) the flexural modulus.

The stress–strain curves of HGM/EP LWTI composites with different diameters and HGM content in a three-point bending test are shown in Figure 2a. The composite’s flexural strength and the slope of the stress–strain curves decreased with the increasing HGM diameter in the four HGM content groups. The HGMs’ strength and modulus values are related to their diameter, which further influenced the reduction of flexural strength and flexural modulus of these lightweight composites. The relationship between HGM diameter and the composites’ flexural strength and flexural modulus is presented in Figure 2b,c.

Figure 2b shows that the flexural strength of the HGM/EP LWTI composites decreased with the increased HGM diameter and volume fraction. For 20 vol.% HGMs, the flexural strength of the composites with 30, 40, and 55 μm diameters were calculated to be 86.45 ± 2.48 MPa, 70.22 ± 1.28 MPa, and 46.98 ± 1.12 MPa, respectively. As the HGM content increased to 50 vol.%, the flexural strength of the composites decreased to 53.83 ± 3.48 MPa, 46.88 ± 1.25 MPa, and 22.34 ± 2.75 MPa, an approximate 37.7%, 33.3%, and 52.5% decrease, respectively, compared with the 20 vol.% samples. According to the stress–strain curves in Figure 2a, the flexural modulus of HGM/EP LWTI composites with different HGM diameters can be calculated, and the results are shown in Figure 2c. The flexural modulus of HGM/EP LWTI composites also decreased as the HGM diameter increased. When the HGM diameter increased from 30 μm to 55 μm, the flexural modulus of the composites decreased by 59.0%, 57.5%, 61.9%, and 66.9%, for the addition of 20 vol.%, 30 vol.%, 40 vol.%, and 50 vol.% HGMs, respectively.

### 3.3. The Thermal Conductivity of HGM/EP LWTI Composites

#### 3.3.1. Thermal Conductivity Model for HGM/EP LWTI Composites

As the HGM volume fraction in the HGM/EP LWTI composites was less than 50 vol.%, the self-consistent approximation method can be used to build a prediction model for thermal insulation performance. The application conditions for the self-consistent approximation method are that the distribution of reinforcement particles in the matrix is continuous and uniform, the reinforcement particles are uniformly surrounded by the matrix, and the entire composite is isotropic [25]. The selected unit is uniformly distributed throughout the entire material, thus, the shape of this unit depends on the shape of the filler particles [26]. Therefore, the assumptions that apply to the material formation process [14,27,28] are listed as follows:

(1) The HGMs, epoxy resin, and the LWTI composites formed using them are all isotropic.
(2) The HGMs are evenly dispersed throughout the resin matrix, and the matrix resin evenly surrounds the surface of the HGMs.
(3) The effect of bubbles on the composite properties is ignored (excluding the hollow interior of the HGMs).

**Figure 3:**
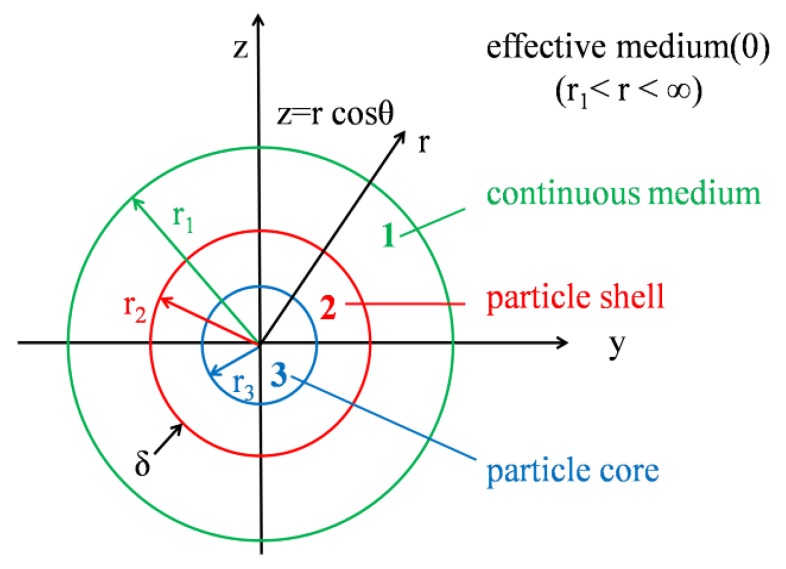
Schematic model of a spherical basic unit.

In this paper, HGM/EP LWTI composites were formed from spherical units composed of epoxy resin uniformly surrounding HGMs. The thermal insulation performance of macro composites is consistent with that of the spherical basic units. Therefore, an analysis of the thermal insulation performance of HGM/EP LWTI composites can be simplified as a spherical unit, as shown in Figure 3.

On the basis of the spherical unit model of HGM/EP LWTI composites, the particle size and wall thickness of the HGMs, the coating resin thickness, and the thermal conductivity of each material should be considered to predict the composites’ thermal insulation performance. A temperature gradient is applied to the HGM/EP LWTI composite spherical unit, and the effective thermal conductivity is calculated by estimating the heat transfer, the average heat flow, and the average temperature gradient in the resin matrix-HGM unit. The symbols appearing in the model derivation are shown in Table S3.

The above spherical units are embedded in homogeneous macro composites by applying a temperature gradient to the macro composites. Therefore, the temperature field distribution can be defined by an independent direction and stable heat conduction. The temperature field in the spherical element can be obtained from the Laplace equation, as shown in Equations (4)–(8):

(5) $$\nabla^{2}T_{n}=\frac{1}{r^{2}}\frac{\partial }{\partial r}\left(r^{2}\frac{\partial T_{n}}{\partial r}\right)+\frac{1}{r^{2}\sin\theta }\frac{\partial }{\partial \theta }\left(\sin\theta \frac{\partial T_{n}}{\partial \theta }\right)=0$$

where n is 0, 1, 2, and 3, which represent the composite material, resin matrix, HGMs, and the gas in the hollow part of the HGMs, respectively.

The temperature distribution of each component in the element can be obtained by solving Equations (4)–(8), as follows:

(6) $$T_{n}=\sum_{m=0}^{\infty }\left(a_{n,m}r^{m}+b_{n,m}r^{-\left(m+1\right)}\right)P_{m}\left(\cos\theta \right)$$

where $P_{m}(\cos\theta )$ is the Legendre polynomial of order M for cosθ, and a and b are arbitrary constants. In an effective medium, an infinite series of orthogonal polynomials tends to terminate at 1; thus, $P_{1}(\cos\theta )=\cos\theta$.

Through the asymptotic temperature field boundary conditions, the following results are obtained:

(7) $$\underset{\left(r\to \infty ,\theta \right)}{\lim}T_{0}\left(r,\theta \right)=-\alpha z=-\alpha r\cos\theta$$

The boundary conditions of contact for each material are ($T_{n}(r,\theta )$) as below,

(8) $$T_{n}\left(r,\theta \right)=\left(A_{n}r+B_{n}r^{-2}\right)\cos\theta$$

where A and B are the temperature distribution coefficients; this formula applies to asymptotic boundary conditions (r→∞). There are six boundaries in the spherical unit model, and each boundary has a coefficient, which is expressed as follows:

(9) $$-k_{n+1}{\left(\partial T_{n+1}/\partial r\right)}_{r_{n+1,}\theta }=-k_{n}{\left(\partial T_{n}/\partial r\right)}_{r_{n+1},\theta }\;(n=0,\;1,\;2)$$

(10) $$=h_{21}\left[T_{2}\left(r_{2},\theta \right)-T_{1}\left(r_{2},\theta \right)\right]\;(n=1)$$

(11) $$T_{n+1}\left(r_{n+1},\theta \right)=T_{n}\left(r_{n+1},\theta \right)\;(n=0,\;2)$$

An algebraic method is used to solve Equations (9)–(11), and the following definitions are introduced:

$${A^{\prime }}_{n}\equiv A_{n}/\alpha \;(n=1,\;2,\;3);\;{B^{\prime }}_{n}\equiv (r_{n}^{-3}/\alpha )B_{n}\;(n=1,\;2);\;{B^{\prime }}_{0}\equiv (r_{1}^{-3}/\alpha )B_{0}$$

The temperature distribution coefficient can be calculated by the following formulas:

(12) $${B^{\prime }}_{0}=\left[-\left(k_{01}-1\right)+3{B^{\prime }}_{1}\right]/\left(2K_{01}+1\right)$$

(13) $${A^{\prime }}_{1}=-\left[3k_{01}+2\left(k_{01}-1\right){B^{\prime }}_{1}\right]/\left(2K_{01}+1\right)$$

(14) $${B^{\prime }}_{1}=3\left\{\left[-\left(\beta_{2}^{-1}+1\right)+k_{21}\right]{\left(r_{2}/r_{3}\right)}^{3}\left(K_{32}+2\right)-\left[\left(2\beta_{2}^{-1}-1\right)-2k_{21}\right]\left(k_{32}-1\right)\right\}/D$$

(15) $${A^{\prime }}_{2}=-9\upsilon_{f}^{-1}\left(k_{32}+2\right)/D$$

(16) $${B^{\prime }}_{2}=9\upsilon_{f}^{-1}\left(k_{32}-1\right)/D$$

(17) $${A^{\prime }}_{3}=-27\upsilon_{f}^{-1}{\left(r_{2}/r_{3}\right)}^{3}/D$$

where

(18) $$\begin{array}{ll} D= & -\left[\left(2k_{01}+1\right)-2\upsilon_{f}\left(k_{01}-1\right)\right]\left(k_{32}-1\right)\\ & \times \left[2-\beta_{2}\left(2k_{21}+1\right)\right]/\left(v_{f}\beta_{2}k_{01}\right)+3\left(k_{32}-1\right)\\ & \times \left[2\left(2k_{01}+1\right)-\beta_{2}\left(2k_{01}+1\right)\right]/\left(v_{f}\beta_{2}k_{01}\right)\\ & +{\left(r_{2}/r_{3}\right)}^{3}\left(k_{32}+2\right)\{\left[2+\beta_{2}\left(k_{21}+2\right)\right]\left(2k_{01}+1\right)\\ & +2v_{f}\left[1-\beta_{2}\left(k_{21}-1\right)\right]\left(k_{01}-1\right)\}/\left(v_{f}\beta_{2}k_{01}\right) \end{array}$$

where

(19) $$k_{mn}=k_{m}/k_{n}$$

(20) $$\beta_{2}=h_{21}r_{2}/k_{2}$$

(21) $$v_{f}={\left(r_{2}/r_{1}\right)}^{3}=\mathrm{volume}\;\mathrm{fraction}\;\mathrm{of}\;\mathrm{HGMs}$$

The distribution of temperature gradient and heat flow follows the basic thermodynamic equation.

(22) $$H\equiv -\nabla T={\hat{\varepsilon }}_{i}H_{i}$$

(23) $$q\equiv k_{eff}H={\hat{\varepsilon }}_{i}q_{i}$$

where ${\hat{\varepsilon }}_{i}$ presents the unit vector in the i direction; $K_{eff}\equiv k_{0}$.

From the temperature distribution results for the spherical unit, the effective thermal conductivity can be obtained as follows:

(24) $$k_{eff}={\bar{q}}_{i}/{\bar{H}}_{i}$$

where the horizontal line is the average value within the unit, and only the z-axis heat flow is non-zero owing to the symmetry, which can be expressed as below:

(25) $${\bar{q}}_{z}={\bar{q}}_{z,1}v_{1}+{\bar{q}}_{z,2}v_{2}+{\bar{q}}_{z,3}v_{3}$$

(26) $${\bar{q}}_{z,n}=k_{n}{\bar{H}}_{z,n}$$

where

(27) $${\bar{H}}_{z}=H_{z}^{0}={\bar{H}}_{z,1}v_{1}+{\bar{H}}_{z,2}v_{2}+{\bar{H}}_{z,3}v_{3}+J_{z}^{\left(12\right)}v_{f}$$

*J_z_^(12)^* can be obtained from the interface temperature discontinuity between material 1 and material 2, as follows:

(28) $$J_{z}^{\left(12\right)}=\left(A_{2}-A_{1}\right)+\left(B_{2}-B_{1}\right)r_{2}^{-3}$$

From Equations (26) and (27), the expression of heat flow in the z-axis direction can be obtained as follows:

(29) $${\bar{q}}_{z}=k_{eff}H_{z}^{0}=k_{1}{\bar{H}}_{z,1}v_{1}+k_{2}{\bar{H}}_{z,2}v_{2}+k_{3}{\bar{H}}_{z,3}v_{3}$$

According to Equation (29), the effective thermal conductivity of the composites is deduced as

(30) $$k_{eff}=\frac{k_{1}{\bar{H}}_{z,1}v_{1}+k_{2}{\bar{H}}_{z,2}v_{2}+k_{3}{\bar{H}}_{z,3}v_{3}}{{\bar{H}}_{z,1}v_{1}+{\bar{H}}_{z,2}v_{2}+{\bar{H}}_{z,3}v_{3}+J_{z}^{\left(12\right)}v_{f}}$$

Combining Equation (27) and Equation (30),

(31) $$k_{eff}=k_{1}+\left(k_{2}-k_{1}\right)\frac{{\bar{H}}_{z,2}}{H_{z}^{0}}v_{2}+\left(k_{3}-k_{1}\right)\frac{{\bar{H}}_{z,3}}{H_{z}^{0}}v_{3}-k_{1}\frac{J_{z}^{\left(12\right)}}{H_{z}^{0}}v_{f}$$

The internal temperature distribution for each material is given by the following:

(32) $$T_{n}\left(r,\theta \right)=\left(A_{n}r+B_{n}r^{-2}\right)\cos\theta$$

The corresponding temperature gradient in the Z direction can be written as follows:

(33) $$-H_{zn}=\frac{\partial T_{n}}{\partial z}=A_{n}+\left(\sin^{2}\theta -2\cos^{2}\theta \right)r^{-3}B_{n}$$

The average temperature gradient of the nth material is as follows:

(34) $${\bar{H}}_{z,n}=-A_{n}$$

Finally, the following simple thermal conductivity formula is derived:

(35) $$k_{eff}=\left[\frac{2\left(1-v_{f}\right)F-3\beta_{2}\phi }{\left(2+v_{f}\right)F-3\beta_{2}\phi }\right]k_{1}$$

where *F* and ϕ are obtained from Equations (36) and (37).

(36) $$F\equiv \left(k_{32}-1\right)\left[2-\beta_{2}\left(2k_{21}+1\right)\right]+\left(k_{32}+2\right)\times \left[1-\beta_{2}\left(k_{21}-1\right)\right]{\left(r_{2}/r_{3}\right)}^{3}$$

(37) $$\phi =2\left(1-{\left(r_{2}/r_{3}\right)}^{3}\right)k_{21}-\left(2+{\left(r_{2}/r_{3}\right)}^{3}\right)k_{31}$$

From the relationship between the wall thickness and the inner and outer HGM diameters, the following formula is obtained:

(38) $$r_{3}=r_{2}-\delta$$

According to the filler shape and structure, the above formula for calculating the thermal conductivity can be further simplified. For the HGMs used in our study, the contact thermal resistance $\beta_{2}\to \infty$, and then the known parameters of the epoxy resin matrix, the wall thickness, and the hollow portion of the HGMs, are introduced into Equations (35)–(37). The expression for the effective thermal conductivity of HGM/EP LWTI composites is simplified as follows:

(39) $$k_{eff}=\left[\frac{\left(1+2{\left(r_{2}/\left(r_{2}-\delta \right)\right)}^{3}\right)\left(1-v_{f}\right)+3.31\left(1+2v_{f}\right)\left({\left(r_{2}/\left(r_{2}-\delta \right)\right)}^{3}-1\right)}{\left(2+v_{f}\right)\left(1+2{\left(r_{2}/\left(r_{2}-\delta \right)\right)}^{3}\right)+6.62\left(1-v_{f}\right)\left({\left(r_{2}/\left(r_{2}-\delta \right)\right)}^{3}-1\right)}\right]*0.452$$

The above model can be used to predict the thermal insulation performance of lightweight thermal insulation composites by adjusting the content, particle size, and wall thickness of the HGMs. In this study, the effective thermal conductivity of HGM/EP LWTI composites is obtained by introducing the parameters of the EP and HGMs into the formula, as shown in Table 2. The prediction results show that the thermal conductivity of HGM/EP LWTI composites declines noticeably with the increased HGM particle size and volume fraction.

#### 3.3.2. The Experimental Thermal Conductivity Results

**Figure 4:**
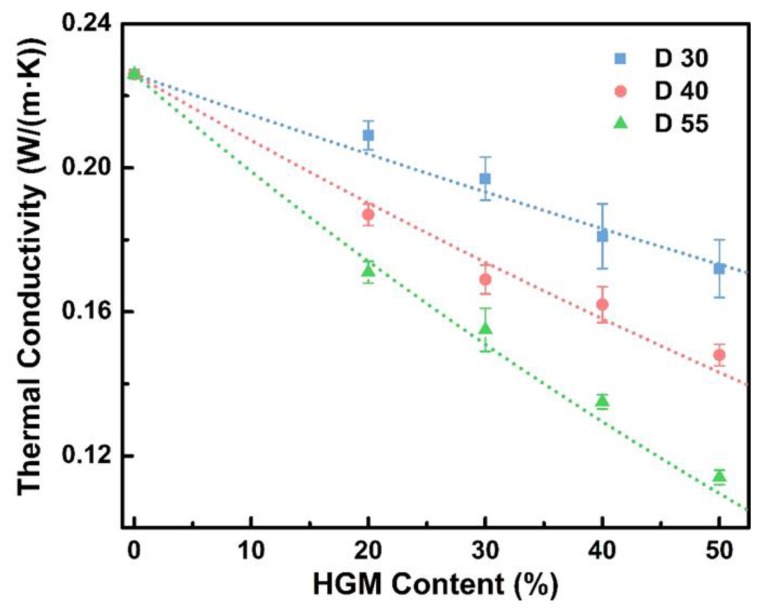
The thermal conductivity calculation of three-phase lightweight composites and actual test values.

Further tests were carried out to characterize the effect of HGMs on the thermal conductivity of HGM/EP LWTI composites with different HGM diameters and content, as shown in Figure 4 (solid dots). As expected, the thermal conductivity of HGM/EP LWTI composites decreased with the increasing HGM diameter, when the HGM content in the composites was the same. By increasing the HGM diameter from 30 µm to 55 µm, the thermal conductivity of the HGM/EP LWTI composites decreased from 0.209 W/m·K to 0.171 W/(m·K) for composites with 20 wt.% HGMs and from 0.197 W/(m·K) to 0.155 W/(m·K) for composites with 30 wt.% HGMs, that is, decreases of 18.4% and 21.5%, respectively. For the lightweight composite with 40 wt.% HGMs, the thermal conductivity decreased from 0.181 W/(m·K) to 0.135 W/(m·K) as the diameter of HGMs varied from 30 µm to 55 µm. In addition, the thermal conductivity of these samples decreased significantly from 0.172 W/(m·K) to 0.121 W/(m·K) as the HGM diameter increased from 30 µm to 55 µm, a decrease of 29.6%. The larger the HGM diameter, the more gas in each HGM, and the fewer HGMs existing in the composites. This means that the gas distribution characteristic is large, but the total amount of gas is lower. Conversely, as the HGM diameter decreases, the total gas in the HGM/EP LWTI composites will be larger in volume, but more broadly distributed. The thermal conductivities of HGMs and EP are much higher than that of the gas inside HGMs, and the heat conduction path for smaller-diameter HGMs is shorter than that of larger-diameter HGMs. Thus, thermal resistance of smaller-diameter HGMs is lower than that of larger-diameter HGMs, the thermal conductivity of HGM/EP LWTI composites decreases with the increasing HGM diameter and the heat insulation properties will be improved.

Figure 4 also illustrates that the thermal conductivity of HGM/EP LWTI composites decreases with the increasing HGM content of the composites, when the same HGMs are used. The air has a blocking effect on heat transfer because the gas is surrounded by the spherical HGM shell and dispersed uniformly in the EP. As the HGM content increases, the volume of the gas increases, and the heat transfer blocking effect will become more prominent; thus, the thermal conductivity of the composite will decrease, which is consistent with other references [20,29].

Lastly, the effect of HGM content and diameter on the thermal conductivity of HGM/EP LWTI composites was compared through the above experimental data and calculation results from the thermal conductivity prediction model. The results are shown in Figure 4 and Table 3. The colorful lines in Figure 4 compare the experimental data with the theoretical predictions based on the model shown in Equation (39); a good linear fit to the data indicates that effective thermal conductivity of HGM/EP LWTI composites can be predicted well by our three-phase thermal conductivity model. The detailed thermal conductivity values of HGM/EP LWTI composites with different HGM diameters and content are displayed in Table 3. The thermal conductivity calculated by the prediction model agrees well with the test results; all error rates are below 5% and the minimum error is only 0.69%, which matched better than the thermal conductivity models (9.51% maximum relative error) for hollow glass bead-filled polypropylene composites by J. Liang [28] and the effective unit cell approach for alumina/ polyimide composite by Ganapathy et al. (approximately ±5% error rates) [30]. The difference between the numerical calculation results and the measured results may be owing to the bubble residual or insufficient mixing during the preparation of the HGM/EP LWTI composites. However, the predicted results still match the actual data well because the resin matrix, the wall thickness, the HGM particle size, and other parameters (such as air) were fully considered during the derivation of this three-phase thermal conductivity model. Thus, this three-phase thermal conductivity model can be used in the theoretical simulation of the thermal conductivity of epoxy resin-based lightweight thermal insulation composites, and serve as a theoretical basis for the study and design of these types of polymer-based, thermally insulated composite materials.

### 3.4. Insulation Mechanism of HGM/EP LWTI Composites

**Figure 5:**
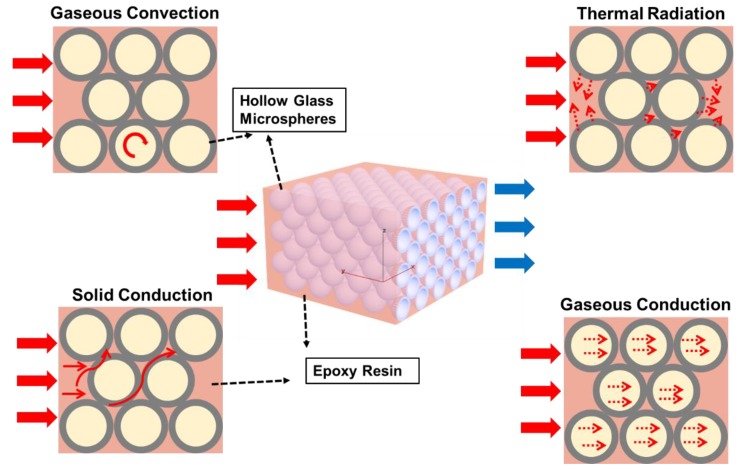
A schematic of thermal transport pathways in HGM/EP LWTI composites.

The thermal conductivity of insulating materials is influenced by many factors such as conduction, convection, radiation, and various other factors in practical application. According to the second law of thermodynamics, heat transfer will occur as long as there is a temperature difference in the system [31]. The mechanism of heat insulation materials is blocking the heat transfer and slowing the heat exchange [32]. In this paper, HGM/EP LWTI composites are a three-phase composite material, including the resin matrix phase, the spherical shell of HGMs, and the gas phase inside the HGMs. When heat is transferred in the EP matrix of LWTI composites, part of the heat is transferred by the EP and another part is transferred by the HGMs. Owing to the low thermal conductivity of HGMs, the heat conduction path in HGM-filled composites is longer, the heat blocking is greater, and the heat insulation performance is better. Generally speaking, heat transport in HGM-filled EP LWTI composite materials includes three main methods [7,26], as shown in Figure 5: (1) gaseous convection within the HGMs; (2) thermal radiation on the surface of the HGMs; and (3) solid and gaseous conduction.

#### 3.4.1. The Gaseous Convection within the HGMs

HGMs are closed hollow materials with gas sealed inside their shells, and their convection performance is affected by their size and thickness. The HGM thickness in this study is small, which prevents convection of the gas inside the HGMs effectively. Thus, the HGM particle size plays a key role in gaseous convection flow resistance. Because the HGM diameter is small, the resistance to gaseous flow is very high; gaseous convection will not occur. The gaseous convection in HGMs can be obtained by Jeffery’s formula.

(40) $$L=\frac{ga(\theta_{1}-\theta_{2})d^{3}}{H\gamma }$$

(41) $$H=\frac{\lambda }{\rho c}$$

where H is the gas thermal diffusion coefficient, λ is the gas thermal conductivity, ρ is the gas density, c is the gas specific heat capacity, a is the gas expansion coefficient, d is the HGM diameter, γ is the gas kinematic viscosity, and $\theta_{1}-\theta_{2}$ is the temperature difference between the two sides of the HGMs.

Jeffery’s formula shows that the larger the HGM diameter, the larger the L. Conversely, it is difficult to have gaseous convection if the diameter of HGMs is small, and thermal conductivity is also lower. The HGM diameters used in this study were substituted into (40) and (41). As the diameters of only a few microns were far less than the required size for convection, the convection contribution is negligible.

#### 3.4.2. The Thermal Radiation on the Surface of the HGMs

The walls of the HGMs are really thin—only several micrometers. Furthermore, the absorption of thermal radiation by the EP matrix is much stronger than that of HGMs. Thus, the proportion of thermal radiation on the surface of the HGMs in the whole heat transfer progress is very small, and the influence of thermal radiation on the HGM/EP LWTI composites is relatively low. Therefore, the thermal radiation on the surface of HGMs can also be neglected for HGM/EP LWTI composites in this paper.

#### 3.4.3. The Solid and Gaseous Conduction

The heat transport in the HGM/EP LWTI composites is mainly governed by solid and gaseous conduction in this study. Solid conduction occurs in the EP matrix and the wall of HGMs. The heat conduction path becomes longer after the addition of HGMs, which is beneficial to reducing the thermal conductivity of composite materials. The gaseous conduction occurs through the gas in the HGMs. The gas conductivity in the HGMs is near 0; thus, HGMs block the path of heat conduction, and the absorbed heat conducted slowly from one side to another. Therefore, the HGM/EP LWTI composites present low thermal conductivity and good thermal insulation performance.

## 4. Conclusions

In summary, the relationship between the density, diameter, and strength of HGMs in HGM/EP LWTI composites was studied first to investigate the basic composite material performance. As expected, the density, the flexural strength, and the flexural modulus will be decreased with the increasing HGM diameter and volume fraction. The flexural strength and the flexural modulus of HGM/EP LWTI composites could still be as high as 22.34 ± 2.75 MPa and 1.34 ± 0.03 GPa, respectively, while the density was only 0.591 g/cm^3^. Then, we presented a three-phase prediction model for the effective thermal conductivity by considering the resin matrix, the wall thickness, the HGM particle size, and other parameters (such as air) fully. The prediction results of our HGM/EP LWTI composites were further compared with experimental data, showing that this three-phase thermal conductivity model provides accurate thermal conductivity prediction of HGM/EP LWTI composites with different HGM diameters and content. All error rates of the experimental data and the theoretical predictions were below 5% and the minimum error was only 0.69%. Lastly, the heat transport in the HGM/EP LWTI composites is mainly governed by solid and gaseous conduction in this study, and the HGM/EP LWTI composites presented low thermal conductivity and good thermal insulation performance owing to the effective blocking of heat conduction by HGMs. More importantly, this work presents insights into this type of hollow sphere-filled material design through parametric studies; the prediction model and method are also easy to use to predict the thermal conductivity of other hollow sphere-filled materials.

## Figures and Tables

**Table 1:** The relationship of porosity with different hollow glass microsphere (HGM) diameter and contents.

Diameter (μm)	Content of HGMs (vol.%)	*ρ_th_* (g/cm^3^)	*ρ_exp_* (g/cm^3^)	*φ_v_* (%)
30	20	1.071	1.045	2.41
	30	1.012	0.979	3.28
	40	0.953	0.912	4.33
	50	0.895	0.845	5.50
40	20	1.005	1.027	2.17
	30	0.946	0.915	3.33
	40	0.865	0.831	4.03
	50	0.785	0.743	5.34
55	20	0.981	0.930	5.18
	30	0.877	0.826	5.86
	40	0.773	0.709	8.39
	50	0.670	0.591	11.71

**Table 2:** Predicted thermal conductivity for the prepared HGM/epoxy resin (EP) lightweight thermal insulation composites.

Thermal Conductivity (W/(m·K))	Volume Fraction			
	20%	30%	40%	50%
D30	0.204	0.193	0.183	0.173
D40	0.190	0.174	0.158	0.143
D55	0.174	0.151	0.129	0.110

**Table 3:** A comparison of experimental data and calculated results from the thermal conductivity prediction model for HGM/EP LWTI composites.

HGMs Content (vol.%)	Type	Thermal Conductivity Theoretical Value (W/(m·K))	Thermal Conductivity of Actual Value (W/(m·K))	Error (%)
20	D30	0.204	0.209	2.532
	D40	0.190	0.187	1.710
	D55	0.174	0.171	1.755
30	D30	0.193	0.197	1.918
	D40	0.174	0.169	2.738
	D55	0.151	0.155	2.679
40	D30	0.183	0.181	1.137
	D40	0.158	0.162	2.474
	D55	0.129	0.135	4.246
50	D30	0.173	0.172	0.685
	D40	0.143	0.148	3.363
	D55	0.110	0.114	4.090

## Supplementary Materials

The following are available online at https://www.mdpi.com/2073-4360/12/3/518/s1, Table S1: The properties of HGMs; Table S2: Constitutive design of HGM/EP LWTI composites; Table S3: Symbols in model derivation.
