# Supplementary material for: Investigation of the Thermal Conductivity of Resin-Based Lightweight Composites Filled with Hollow Glass Microspheres
Source: Polymers (Basel). 2020 Feb 29;12(3):518. doi: 10.3390/polym12030518 (PMC7182894; doi:10.3390/polym12030518)
Supplement: Supplementary file 1 [file polymers-12-00518-s001.pdf]

Supporting Information

Investigation of the thermal conductivity of resin-based lightweight composites filled with hollow glass microspheres

Zhipeng Xing, Hongjun Ke, Xiaodong Wang*, Ting Zheng*, Yingjie Qiao, Kaixuan Chen, Xiaohong Zhang, Lili Zhang, Chengying Bai, Zhaoran Li

School of Material Science and Chemical Engineering, Harbin Engineering University, Harbin 150001, China.

Corresponding author: wangxiaodong@hrbeu.edu.cn (X.Wang);

tingzheng@hrbeu.edu.cn (T. Zheng)

Table S1 The properties of HGMs

Type	Diameter /μm	Density /g·cm^{-3}	Compressive strength /MPa
D30	30	0.60	68.9
D40	40	0.38	30.0
D55	55	0.15	2.1

Table S2 Constitutive design of HGM/EP LWTI composites

HGMs /vol.%	E-51 /vol.%	Hardener /vol.%	HGM Diemater		
			30 μm	40 μm	55 μm
20%	64%	16%	D30-20	D40-20	D55-20
30%	56%	14%	D30-30	D40-30	D55-30
40%	48%	12%	D30-40	D40-40	D55-40
50%	40%	10%	D30-50	D40-50	D55-50

Table S3 Symbols in model derivation

Symbols	Physical Meaning	Unit
T	Temperature	°C
r	Polar Radius	μm
θ	Polar Angle	
α	Temperature Gradient in Composite	
h_{21}	Thermal Resistance of the Interface Contact between HGMs and Matrix resin	$(m^2 \cdot K)/W$
v_n	the Volume Fraction of the nth Material in the Unit	
β_n	Contact Thermal Resistance	$(m^2 \cdot K)/W$
v_f	the Volume Fraction of HGMs	
δ	the Wall Thickness of HGMs	m
q	Heat Flow	(W/m^2)
